# Impact of Gene Molecular Evolution on Phylogenetic Reconstruction: A Case Study in the Rosids (Superorder Rosanae, Angiosperms)

**DOI:** 10.1371/journal.pone.0099725

**Published:** 2014-06-16

**Authors:** Khidir W. Hilu, Chelsea M. Black, Dipan Oza

**Affiliations:** Department of Biological Sciences, Virginia Tech, Blacksburg, Virginia, United States of America; University of Georgia, United States of America

## Abstract

Rate of substitution of genomic regions is among the most debated intrinsic features that impact phylogenetic informativeness. However, this variable is also coupled with rates of nonsynonymous substitutions that underscore the nature and degree of selection on the selected genes. To empirically address these variables, we constructed four completely overlapping data sets of plastid *matK*, *atpB*, *rbcL*, and mitochondrial *matR* genes and used the rosid lineage (angiosperms) as a working platform. The genes differ in combinations of overall rates of nucleotide and amino acid substitutions. Tree robustness, homoplasy, accuracy in contrast to a reference tree, and phylogenetic informativeness are evaluated. The rapidly evolving/unconstrained *matK* faired best, whereas remaining genes varied in degrees of contribution to rosid phylogenetics across the lineage's 108 million years evolutionary history. Phylogenetic accuracy was low with the slowly evolving/unconstrained *matR* despite least amount of homoplasy. Third codon positions contributed the highest amount of parsimony informative sites, resolution and informativeness, but magnitude varied with gene mode of evolution. These findings are in clear contrast with the views that rapidly evolving regions and the 3^rd^ codon position have inevitable negative impact on phylogenetic reconstruction at deep historic level due to accumulation of multiple hits and subsequent elevation in homoplasy and saturation. Relaxed evolutionary constraint in rapidly evolving genes distributes substitutions across codon positions, an evolutionary mode expected to reduce the frequency of multiple hits. These findings should be tested at deeper evolutionary histories.

## Introduction

Genomic regions vary considerably in their potential phylogenetic informativeness and subsequent contribution to resolving a given set of taxa over specified time points. Among the properties inherent to genomic regions, rate of nucleotide substitution is regarded to have a profound impact in phylogenetic reconstruction [1]–[8]. This has led to a debate over the “optimal” rate of substitution for a choice genomic region within a contextual historic depth [1], [2], [6], [9]–[15]. Rate of substitution in this ideal genomic region should not be too low to generate inadequate signal or too high to inflate noise. Consequently, the prevalent approach promotes the use of rapidly evolving regions in shallow evolutionary histories and slowly evolving regions for deeper epochs [3], [16]–[20]. The exclusion of rapidly evolving regions from deep phylogenetic reconstruction is generally based on the premise that multiple hits confounded by extended time scale could be significant enough to conceal phylogenetic signals and elevate homoplasy, with saturation reaching levels that can negatively impact tree structure [9], [11], [14], [15]. It was further suggested that accumulation of multiple hits in rapidly evolving regions can obscure potential synapomorphies and may also result in long-branch attraction [6], [21], [22]. However, opposing points have been raised that promote the effectiveness of rapidly evolving and less constrained genomic regions in deep-level phylogenetics [2], [13], [23]–[26].

Similarly, the 3^rd^ codon position was down weighted or excluded in phylogenetic analyses due to higher rates of substitution compared with the 1^st^ and 2^nd^ positions [27]–[33]. It has been argued [22] that the greater average of substitution rate for 3^rd^ codon positions might reduce phylogenetic signal due to long-branch attraction in parsimony reconstructions. Li and Graur [34] indicated that unlike 1^st^ and 2^nd^ codon positions, the 3^rd^ codon position is highly saturated and contributes higher levels of homoplasy. However, these views have been disputed [2], [10], [35]–[43]. The 3^rd^ codon position is under relaxed selection since mutations in it are less likely to lead to nonsynonymous substitutions. Theoretically, mutations in the 1^st^, 2^nd^ and 3^rd^ codon positions translate into 96%, 100%, and 31% nonsynonymous substitutions, respectively [34].

These opposing notions have not been tested within a well-defined experimental design. We evaluate here the overall phylogenetic informativeness of four protein-coding genes (*rbcL*, *atpB*, *matK*, *matR*) with contrasting combinations of mode and tempo of evolution in the rosids lineage (angiosperms) using PhyDesign [6] in conjunction with various statistical measures. The *matR* gene is mitochondrial whereas the others are plastid genes. Functionally, *matK* and *matR* are group II intron maturases, *rbcL* codes for the large subunit of RuBisCo, an essential photosynthesis enzyme, while *atpB* encodes the beta subunit of the plastid ATPase [44]–[46]. Substitution rate in *matK* is about twice that of *rbcL*, and 6.5 times at nonsynonymous sites [25], [47]. The synonymous substitution rate in *matR* is approximately four times slower than those of *rbcL* and *atpB*, whereas the rate of nonsynonymous mutations is considerably higher [3], [48], [49]. The substantially higher rates of nonsynonymous mutations in *matR* and *matK* imply reduced selectional constraints compared with *rbcL* and *atpB* [3], [25], [46], [48], [50]. Thus, the four genes represent spectra of nucleotide substitution rates from the rapidly evolving *matK* to *rbcL*, *atpB*, and *matR*, and amino acid substitution rates from *matR* to *matK*, *rbcL*, and *atpB*. For convenience, we use the terms *rapidly* and *slowly* evolving for overall rates of substitutions and *constrained* and *unconstrained* for degrees of nonsynonymous substitution.

**Figure 1:**
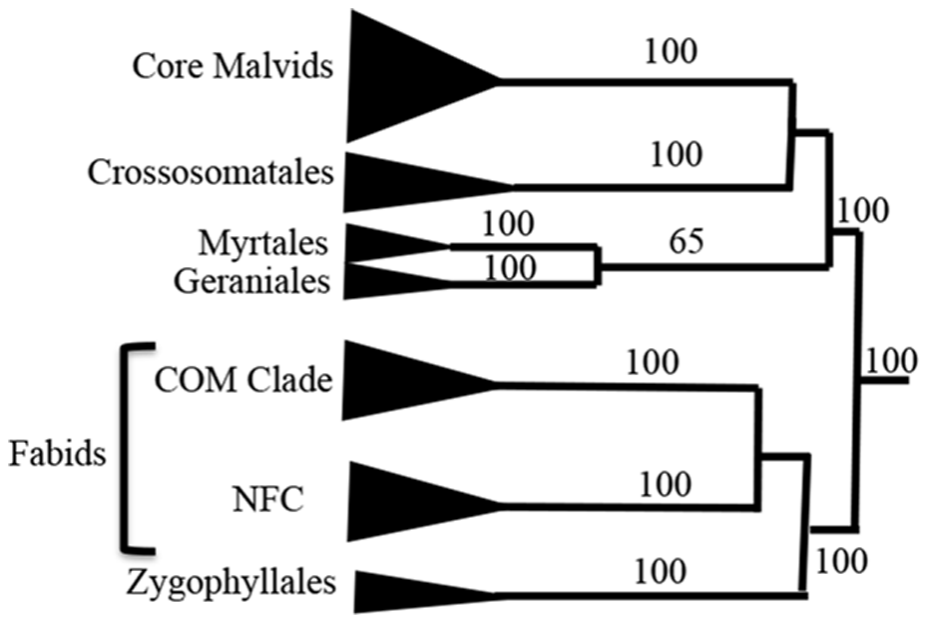
A summarized backbone relationships among the rosids lineages based on Wang et al. [19] phylogenetic study. A total evidence maximum likelihood rosid tree redrawn from Wang et al. (2009) with percent bootstrap support values ≥50% noted on branches. COM: Celastrales, Malpighiales, and Oxalidales. NFC (Nitrogen Fixing Clade): Rosales, Fabales, Cucurbitales, and Fagales. The malvids clade includes the basal three orders Myrtales+Geranials/Crossosomatales as sister to the core group.

The choice of the rosids (Superorder Rosanae APG III [51]) is based on current availability of a robust multi-gene phylogeny [19], the documented monophyly of the group with well defined lineages (e.g. rosids, fabids, core malvids), and the detection of rapid radiation in parts of its ∼108 million years (MY) of evolution [19]. The rosid clade, as circumscribed by APG III [52], includes 140 families placed in 18 orders (Vitales placement is uncertain [53]). Two recent rosids-focused phylogenetic studies exist based on four genes [48], and 36 genes plus intervening spacer sequences [19]. The latter study provides the most robust tree for the rosids, and will thus be used as reference tree here. The rosid families fall into two large subclades, Fabidae (fabids) and Malvidae (malvids) (Figure 1). Within the fabids, the Zygophyllales is sister to two clades: the nitrogen fixing clade (NFC; Rosales, Fabales, Cucurbitales, Fagales), and the COM clade (Celastrales, Malpighiales, Oxalidales). The malvids clade includes the historically difficult to place orders Myrtales, Crossosomatales, Geraniales, and Picramniales, in a grade sister to the core malvids (Brassicales, Malvales, Sapindales, Huerteales).

We demonstrate here that the rapidly evolving and unconstrained *matK* provides substantially higher phylogenetic information and structure throughout the rosids history, whereas slowly evolving genes exhibit lower degrees of informativeness regardless of rates of nonsynonymous mutations. The 3^rd^ codon position consistently superseded the 1^st^ and 2^nd^ positions in phylogenetic signal, but the disparity in informativeness is accentuated in the constrained genes. The four genes informativeness profiles combined provide complementary phylogenetic signal across the rosids phylogenetic tree.

## Materials and Methods

### Genomic regions, taxon sampling, and data partitioning

To empirically evaluate the phylogenetic informativeness of four single-copy orthologs selected for this study (plastid *atpB, rbcL*, and *matK*, and mitochondrial *matR*), we generated overlapping data sets of 60 species from the rosid clade. The data set includes newly-generated complete or partial sequences for *matK* and sequences obtained from GenBank (Table S1). The data set covers 43 of the 140 families and represents 16 of the 17 orders currently assigned to the rosid clade *sensu* APG III [51]. The order Rafflesiales sensu APG III [51] is not represented, however, the APG III [51] places the family in the Malpighiales. The Vitaceae (Vitales) is included as part of the core eudicots since its placement in the rosids is equivocal [53], [54]. Taxon sampling was based on achieving strong overlap with the sample used in the Wang et al. [19] study of the rosids. In addition to the rosid taxa, 21 species were included to represent the remaining core eudicot lineages (Solanales, Lamiales, Cornales, Ericales, Caryophyllales, Phyllanthaceae, Berberidopsidales, Vitales, and Dilleniaceae). The data set was partitioned into individual genomic regions and respective codon positions. Trees were rooted in all analyses with representatives of the early diverging eudicots Buxaceae and Trochodendraceae and the first diverging core eudicot Gunneraceae [13], [55]. Information on the species used and sources of the sequences are noted in Table S1.

### Materials, DNA isolation and sequencing

Genomic DNA for the 54 new *matK* sequences was either isolated from material collected in the field or was obtained from various sources (Table S1). Genomic DNA was isolated following Doyle and Doyle [56] procedure as modified in M'ribu and Hilu [57]. The *matK* gene was amplified as described in Crawley and Hilu [8]. Sequencing was performed at the Virginia Bioinformatics Institute at Virginia Tech or Duke University using a Big Dye Terminator Cycle Sequencing Ready Reaction Kit (Applied Biosystems, Foster City, California).

### Sequence alignment and phylogenetic reconstruction

Sequences were manually aligned using the QuickAlign program [58]. Gaps were inserted at the cost of two or more substitutions. However, all data sets were analyzed without the inclusion of gaps as characters in order to avoid character bias among genes since *atpB, rbcL* and *matR* either lack or have few gaps, and to render the data comparable to the Wang et al. [19] study that excluded gaps.

The data sets were analyzed using both Maximum Parsimony (MP) and Maximum Likelihood (RAxML). The MP analyses were conducted in PAUP* version 4.0b [59] and consisted of a heuristic tree search with TBR branch swapping and 1000 random stepwise addition replicates with indels treated as missing data. In the MP analyses of codon partition, the runs did not reach completion for the 1^st^ and 2^nd^ data sets in some of the slowly evolving genes. To avoid subjective inflations in the number of most parsimonious trees and the subsequent impact on consensus tree resolution, we opted to analyze concatenated 1^st^ and 2^nd^ data sets. However, results from individual codon position analyses that reached completion will be noted wherever relevant. A strict consensus tree was generated when multiple most parsimonious trees were recovered. Bootstrap support (BS; Felsenstein [60]) was calculated in PAUP* with 1000 replicates each with 10 random sequence addition replicates using the same conditions as in MP analysis. Due to time constraints, the data sets were analyzed using the Ratchet algorithm [61] as implemented in PRAP2 [62] and executed in PAUP*. The RAxML analyses were conducted in CIPRES portal (http://www.phylo.org) applying the default setting and conducting 1000 replicates. Bootstrap support was calculated for the 50% majority trees. The default Model GTR+I+G was used.

### Measurements of phylogenetic informativeness

PhyDesign (http://phydesign.townsend.yale.edu; [63]) was used to estimate phylogenetic informativeness of genomic regions across rosid evolutionary history to assess their effectiveness in phylogenetic reconstruction in the context of their mode and tempo of evolution. This software program computes the amount of phylogenetic information in a genomic region across the history of the group based on character evolutionary rates. The Townsend [6] metric phylogenetic informativeness has been used effectively in predicting informativeness profiles in various groups [8], [64]–[70]. Although Klopfstein et al. [14] argued that it could lead to overestimation of informativeness in rapidly evolving genes when taxon sampling increased beyond the 4-taxa case, Townsend and Luenberger [71] refuted that notion. Both net and per-site informativeness were computed and contrasted to assess cost-effectiveness of the genes. The concatenated nucleotide data set was partitioned by genes and codon positions. We used these alignments to generate ML trees with RAxML. A fixed age of 108 MY was selected for the rosids divergence, and 6 minimum ages (*Phytolacca*/*Polygonum* 83.5 MY; *Galax*/*Sarracenia* 91.2 MY; *Leea*/*Vitis* 57.9 MY; *Citrus*/*Bursera* 65 MY; *Malpighia*/*Passiflora* 49 MY; *Populus*/*Salix* 48 MY) were designated following Wang et al [19]. The RaxML best tree file and the designated dates were used to reconstruct ultrametric trees in PATHd8 (www.math.su.se/PATHdh; [72]). The ultrametric tree files were executed in MEGA 4.0 [73] to generate a Newick format. These tree files and their corresponding data sets were used as input files in PhyDesign to extract the phylogenetic informativeness for genes and their codon positions.

### Measures of phylogenetic structure and accuracy

Phylogenetic structure encompasses tree resolution and support for depicted relationships. With the focus on the backbone of the rosids (the major clades, their subclades, and the orders), the total number of nodes in a fully resolved tree is 41. We used the number and percentages of nodes resolved in the MP strict consensus tree derived from partitioned (genes and codons) data sets as a measure of resolution. Bootstrap [60] values obtained from RAxML were used as statistical measure of support. Since the Townsend [6] phylogenetic informativeness does not account for homoplasy [63], ensemble consistency index (CI; Kluge and Farris [74]) and ensemble retention index (RI; Farris [75]) were used as measures of homoplasy to evaluate signal vs. noise for the four genomic. Phylogenetic accuracy was assessed by comparing the reconstructed trees from the partition analyses with a model tree for the rosids, namely the total-evidence tree of Wang et al. [19] for incongruences. This latter tree was based on >43,000 base pair (bp) from two nuclear and 34 plastid genes plus some intervening spacers of plastid inverted repeat. It is fully resolved, strongly supported, and topologically highly congruent with relationships recovered in phylogenetic studies on angiosperms, e.g. [24], [55].

### Assessments of molecular evolution and statistical tests

The four protein coding genes display different rates and modes of evolution as reflected in the rates of nonsynonymous substitution and its subsequent impact on amino acid mutations. We estimated the ratio of nonsynonymous substitution per nonsynonymous site to synonymous substitution per synonymous site, dN/dS, using SNAP (www.hiv.lanl.gov; [76]). Statistical testing was carried out in JMP 9 (www.jmp.com). To assess degree of variation in phylogenetic signal at different eras of rosids evolution, the 108 MY time scale was divided into four equal epochs and both net informativeness and its standard deviation for the genes were calculated for each epoch.

## Results

**Figure 2:**
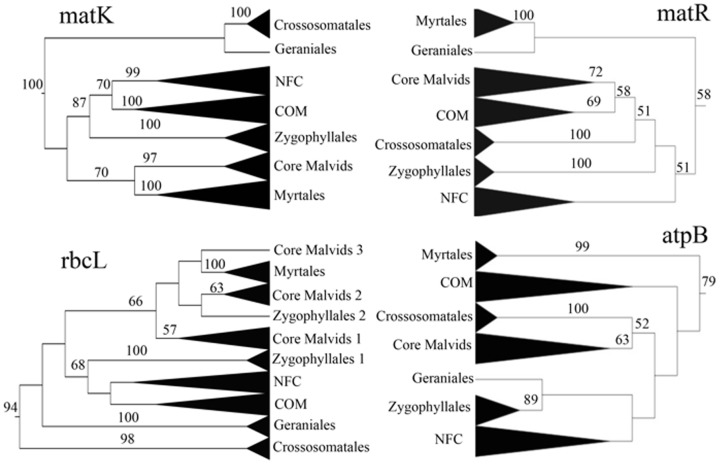
Summaries of the rosids RAxML trees based on the four gene partitions. The backbone relationships among the rosids lineages based on partitioned data sets of *matK*, *matR*, *rbcL*, and *atpB*. Percent bootstrap support values ≥50% are noted. COM: Celastrales, Malpighiales, and Oxalidales. NFC (Nitrogen Fixing Clade): Rosales, Fabales, Cucurbitales, and Fagales. The core malvids group and the Zygophyllales were not recovered as monophyletic lineages in the *rbcL* partition analysis.

**Figure 3:**
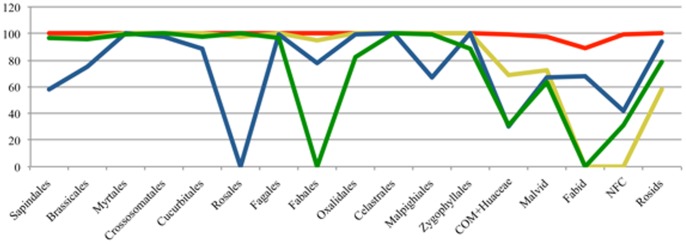
Variation in bootstrap support for the major rosids lineages derived from gene partition analyses. Illustration of the differential bootstrap support for the rosids orders and the major clades calculated from the RAxML analyses based on partitioned gene data sets of *matK* (red), *matR* (yellow), *rbcL* (blue), and *atpB* (green).

**Figure 4:**
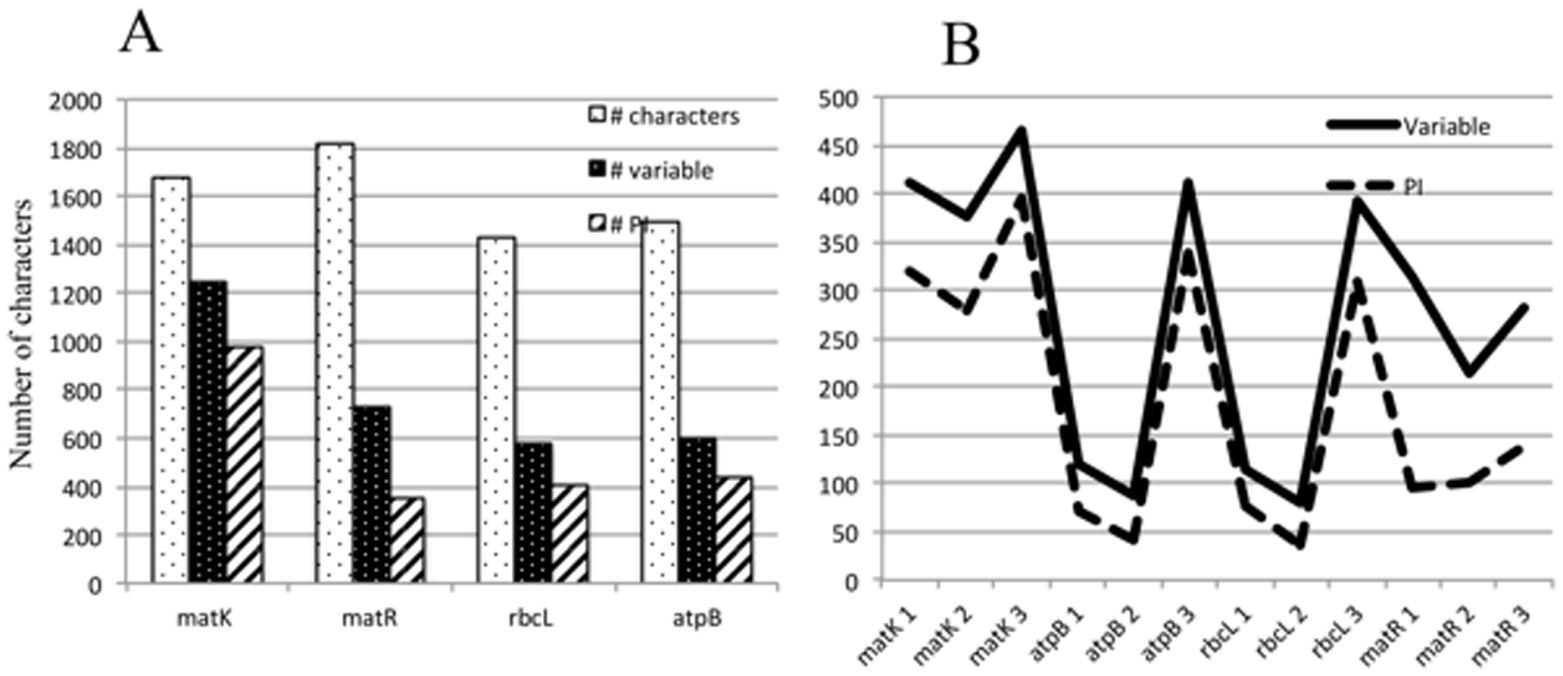
Diagrammatic representation of information on molecular characters from the four gene data sets. The number of total, variable and parsimony informative (PI) characters calculated from the maximum parsimony analyses of *matK*, *matR*, *rbcL*, and *atpB*. (A) gene partitions, (B) the three codon positions partitions (B).

**Figure 5:**
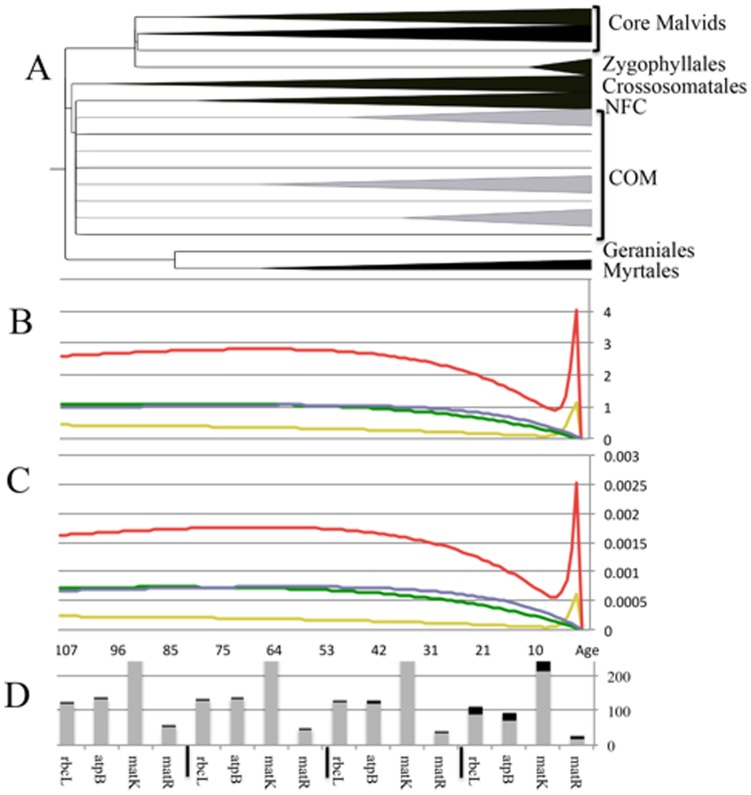
Rosids ultrametric reconstructed in PATHd8 and the informativeness profiles estimated in PhyDesign along with their variability at different epochs of rosids history. (A) Ultrametric tree for the rosids calculated in PATHd8 based on the concatenated four-gene data set. (B) Net informativeness profiles for *matK* (red), *matR* (yellow), *rbcL* (blue), and *atpB* (green) gene partitions; (C) Per-site informativeness profiles for the same genes; (D) The mean (gray) and standard deviation (black) for gene informativeness at four equally divided epochs in the evolutionary history of the rosids. COM: Celastrales, Malpighiales, and Oxalidales. NFC: Nitrogen Fixing Clade, Rosales, Fabales, Cucurbitales, and Fagales. Both *matK* and *matR* display spikes in their informativeness profiles at very recent times.

To be consistent in contrasting phylogenetic tree reconstructed in this study with the reference tree of Wang et al. [19], we will focus on the RaxML trees. However, consensus trees and tree statistics obtained from the MP analyses will also be discussed. The RAxML trees for the four gene partitions are summarized to highlight the major rosid clades (Figures 2–5); the detailed trees are provided in Figures S1–S4.

### Phylogenetic structure

Three tree parameters will be underscored here, namely resolution, node support, and accuracy as compared with the reference tree. Degree of resolution is presented as number and percentage of nodes resolved in the MP strict consensus tree out of a total 41 expected nodes (Table 1). Degree of resolution is highest in *matK* (40, 98%) and lowest in *atpB* (29, 70%). The BS support for 17 major nodes that describe the backbone of the rosids is plotted for analyses based on gene partitions (Figure 3). In the *matK* partition (rapidly evolving/unconstrained), the rosids' monophyly receive 100% BS support (Figure 2), and core malvids and fabids are recovered with 97% and 87% BS support, respectively. Of the three basal malvids orders, the Myrtales appear sister to core malvids (70% BS), whereas the Crossosommatales and Geraniales emerge in a clade sister to remaining rosids (<50% BS). The core malvids structure (Figure S1) is topologically identical to that retrieved in Wang et al. [19]. Within the fabids, Zygophyllaceae emerge as sister to the NFC (99% BS) plus the COM clade (100% BS). Phylogenetic relationships among the orders within the NFC and COM clades differ from those obtained by Wang et al. [19], but incongruences are weakly supported. All orders received 100% BS support (Table S2). The CI and RI values are 0.374 and 0.429, respectively.

**Table 1:** Phylogenetic information and informativeness (net and per-site) of genes and their codon partitions.

Genes/	Character included	Variablesites	PI^a^	CI^b^	RI^c^	MP^d^ node # (%)	Net	Per-site
matK	1635	1250	975	0.374	0.429	40 (98)	2.765	0.187
atpB	1496	600	438	0.39	0.426	29 (71)	0.885	0.074
rbcL	1427	580	402	0.353	0.416	36 (88)	0.912	0.079
matR	1832	732	350	0.692	0.638	33 (80)	0.764	0.021
matK 1/2	1090	789	597	0.400	0.455	40 (98)	0.753/0.596	0.003/0.002
matK 3	545	466	394	0.328	0.435	40 (98)	1.155	0.004
atpB 1/2	998	209	114	0.519	0.478	18^f^ (44)	0.100/0.069	0.000/0.000
atpB 3	498	410	339	0.346	0.438	23 (56)	0.681	0.001
rbcL 1/2	952	195	112	0.414	0.465	15 (37)	0.129/0.061	0.000/0.000
rbcL 3	475	392	308	0.332	0.448	36 (88)	0.571	0.001
matR 1/2	1221	467	216	0.683	0.642	34 (83)	0.203/0.194	0.004/0.000
matR 3	610	281	138	0.604	0.622	23 (56)	0.231	0.001

a: Parsimony Informative.

b: Consistency Index.

c: Retention Index.

d: Maximum Parsimony.

f: Analysis did not reach completion.

Net informativeness could be calculated only for partitioned codon positions individually. Numbers associated with genes refer to codon positions.

The slowly evolving/unconstrained mitochondrial *matR* recovers the rosids (58% BS) as well as the core malvid clade (72% BS), but the fabid clade as traditionally defined [19], [24], [53], [55] is not retrieved (Figure 2). Instead, the COM clade of the fabid appears sister to the malvids, albeit BS support for this relationship is 58%. Two of the early diverging malvids lineages (Myrtales and Geraniales) form a clade sister to all rosids, whereas the third lineage, the Crossosomatales, form along with the Zygophyllales a weakly supported grade sister to the malvids plus COM (Figure 2). All these nodes received 51% BS support at best. The topology of the COM clade is congruent with that of Wang et al. [19], whereas those of the malvid and NFC clades are not (Figure S2). BS support for the monophyly of the orders range from 95–100% (Table S2). The CI and RI values are 0.692 and 0.638, respectively.

The *rbcL* (slow/constrained) data provided 94% BS support for the rosids monophyly and 68% for the fabids. However, core malvids are not monophyletic, with Myrtales and *Tribulus* (Zygophyllaceae) nested in them with 66% BS support, a topology that depicts polyphyletic Zygophyllaceae (Figure 2). The Crossosomatales and Geraniales appear as consecutive sisters to all rosids but their placements receive <50% BS support. The ordinal relationships within the malvid and fabid clades (Figure S3) are incongruent with those obtained by Wang et al. [19], but BS support is weak. Monophyly for the orders, except paraphyletic Rosales, receive 58–100% BS support (Table S2). The CI and RI values are 0.353 and 0.416, respectively.

The *atpB* (slow/constrained) data recover the rosids with 79% BS, but fail to recover the fabids as COM + NFC. Instead, the COM clade diverge after Myrtales as sister to remaining rosids, albeit BS support for this topology is <50%. The Crossosomatales and Geraniales are scattered across the tree (Figure 2, Figure S4). BS Support for the monophyly of the rosids orders is 82–100% (Table S2). The CI and RI values are 0.390 and 0.426, respectively.

### Phylogenetic informativeness of the four genes

Measures of phylogenetic information are based on numbers of PI characters as well as gene informativeness profiles computed in PhyDesign (table 1). The number of PI characters and their proportion out of total characters is lowest for the slowly evolving/unconstrained *matR* (350, 19%) and highest for the rapidly evolving/unconstrained *matK* (975, 58%); the slowly evolving/constrained *atpB* and *rbcL* are intermediate, providing 438 (29%) and 402 (28%) PI characters, respectively (Table 1, Figure 4). Similar trend is also notable in the phylogenetic informativeness estimated in PhyDesign with rapidly evolving *matK* superseding the other genes in net and per-site informativeness across the rosids history (Figure 5). The *matR* gene stands at the lower end of the informativeness spectrum, and *rbcL* and *atpB* are intermediates (Table 1, Figure 5). Rapidly evolving *matK* displays a relative decline in informativeness at deeper histories (≥50MY; (Figure 5). In contrast, net informativeness of the slowly evolving but constrained *rbcL* and *atpB* remain constant at deeper histories but declined in recent epochs (Figure 5). The informativeness profile of *matR* is elevated at deeper epochs than recent ones. The Standard deviation of informativeness for all four genes was quite low across the rosids' history except for the most recent epoch (Figure 5). Curious spikes in the informativeness profiles of unconstrained *matK* and *matR* are notable in modern era; *rbcL* and *atpB* lack these spikes. These spikes are intriguing since both genes evolve under relaxed selection but differ considerably in rates of substitution. However, this phenomenon has been addressed on the PhyDesign website (http://phydesign.townsend.yale.edu/), stating that “those few sites all are estimated to evolve at one very fast rate, leading to a spike that has little biological meaning”. Per-site informativeness profiles followed the same trend as net informativeness profiles in all four genes (Figure 5).

### Phylogenetic informativeness at codon partitions

**Figure 6:**
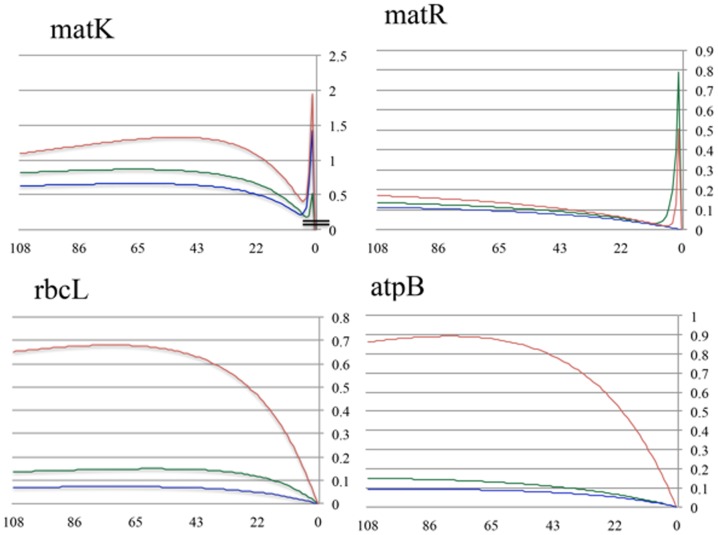
Informativeness profiles for codon positions measured in PhyDesign. Net informativeness profiles for 1^st^ (green), 2^nd^ (blue) and 3^rd^ (red) codon positions of *matK*, *matR*, *rbcL*, and *atpB* computed in PhyDesign. Note the informativeness values for *matK* codon positions starting at 0.5, highlighted by double lines on the y-axis. Both *matK* and *matR* exhibit spikes in their informativeness profiles at very recent times.

The 3^rd^ codon positions account for larger proportions of PI characters compared with their respective 1^st^ and 2^nd^ positions, but degree of disparity varied with gene mode of evolution (Figure 4; Table 1). Net and per-site informativeness was highest in the 3^rd^ codon positions, followed by the 1^st^ codon position, except for the per-site informativeness in *matR* where it is highest in the 1^st^ codon position followed by the 3^rd^ (Table 1). Overall, the three codon partitions of *matK* exhibit higher magnitudes of PI characters than corresponding codon positions of the other three genes (Table 1, Figure 4). Both unconstrained *matK* and *matR* display more uniformity in number of PI characters across codon positions with standard deviation (SD) being 59 and 23, respectively. This is juxtaposed with *atpB* and *rbcL* where the number of PI characters are disproportionally skewed towards the 3^rd^ codon positions (SD  = 164 and 147, respectively) (Table 1, Figure 4). Codon position informativeness calculated in PhyDesign varies with gene mode than tempo of evolution, mirroring the patterns noted in the PI data (Table 1; Figure 6), with *matR* showing the highest uniformity among codon positions (SD  = 0.019). The 3^rd^ codon position profile of *matK* experienced a relatively higher degree of decline in informativeness at deeper histories (≥50 MY; Figure 6). When contrasted with slight decline displayed by the 3^rd^ codon positions of *atpB* and *rbcL* (≥85 and 75 MY ago, respectively). The informativeness profiles of all three codon positions in *matR* remain elevated at deeper rosids history (Figure 6). Similar recent spikes in informativeness profiles are evident in *matK* and *matR* codon positions.

In the phylogenetic analyses of codon partitions, degrees of resolution are comparable among trees based on the individual codon partitions in the unconstrained *matK* and *matR*, but increase resolution is skewed toward the 3^rd^ codon position in the constrained *rbcL* and *atpB* (Table 1). In *matR*, the 3^rd^ codon position provides less tree resolution than the 1^st^ and 2^nd^ (Table 1).

### Phylogenetic noise

We used ensemble CI and RI values as measures of homoplasy. PhyDesign does not profile phylogenetic noise or provide estimates of overall noise for gene or codon partitions. The values for the CI and RI measures are highly correlated (r^2^ = 0.938, F_1,16_ = 212, P<0.0001) throughout the partitioned analyses of genes and codon positions. Consequently, we arbitrarily opted to use the CI values. Homoplasy (1-CI) was lowest in *matR*, whereas it is quite comparable for the other three genes despite striking difference in rates of nucleotide and amino acid substitutions (Table 1). Homoplasy is highest in the 3^rd^ codon position and lowest in the 2^nd^ for all four genes. Again, homoplasy is lowest in *matR* for all three codon positions (Table 1). Homoplasy in the 3^rd^ codon position is quite similar for *matK*, *atpB* and *rbcL* (0.672, 0.654 and 0.668, respectively) despite marked differences in rates of substitutions.

## Discussion

### Gene informativeness

The disparity in tempo of gene evolution is reflected by the marked differences in the proportions of variable characters, ∼40% in *rbcL*, *atpB* and *matR* compared with 75% in *matK* (Table 1, Figure 4). However, the likelihood of a variable character to be parsimony informative was comparable (*X^2^* = 6.591, *P* = 0.037) among the rapidly evolving/unconstrained *matK* and the slowly evolving/constrained *rbcL* and *atpB* (78% vs. 69% and 73%, respectively). As a consequence, the 2.2–2.4 folds in number of PI characters contributed by *matK* compared with *rbcL* and *atpB* could be ascribed in part to the availability of a larger pool of variable characters in *matK* (Table 1, Figure 4). In a marked contrast, the slowly evolving but unconstrained *matR* exhibits the lowest proportion of PI characters (48%) despite its similarity to the other two slowly evolving genes, *rbcL* and *atpB*, in proportion of variable characters. This pattern may imply lower probability for a *matR* variable characters to be translated into PI characters in the rosids.

Net informativeness of *matK* is 2.6 fold those of *atpB* and *rbcL*, and 7.8 that of *matR* (Table 1). A strong correlation (r^2^ = 0.994, F_1,4_ = 329, P<0.003) is found between the per-site informativeness and the number of PI characters for the four genes (Table 1, Figure 5). These two measures together provide a reliable estimation of phylogenetic signal in the rosids, and point to *matK* as being by far the most informative gene across the evolutionary history of the rosids compared with the three slowly evolving genes (Figure 5). The high performance of *matK* is likely due to expanding character state choices (character-state space [77]) and subsequent minimization of the impact of homoplasy [43], [77]–[79]. In the rosids, the degree of substitution in the 3^rd^ codon position in *matK* is quite comparable to those in the 3^rd^ codon positions of *atpB* and *rbcL* (466 vs. 410 and 392, respectively, Fig. 4B). Should this rapidly evolving *matK* be as evolutionarily conserved as *rbcL* and *atpB*, the anticipated skewed substitution rate toward its 3^rd^ codon position would augment the likelihood of multiple hits and homoplasy due to an expected 41% surge in nucleotide mutations in its 3^rd^ codon positions (calculated from Table 1). Therefore, superimposing elevation in rate of nonsynonymous mutations on the overall accelerated rate of substitution in *matK* helps in reducing the mutation load on its 3^rd^ codon position. Mossel and Steel [80], Steel and Penny [81] and Townsend et al. [15] have demonstrated that increased accessibility of characters to multiple states reduces the potential noise stemming from multiple hits. The decline in the informativeness profiles of the *matK's* 3^rd^ codon position from the rosids crown group divergence to ∼50 MY ago is probably a function of its substitution rate. Despite this decline, its overall informativeness and the profile remain above those of the other two codon positions (Figure 6; Table 1).

The informativeness profile of *matK* declined deep in rosids history (≥50 MY ago) in contrast with that of *matR* (Figure 5); both are evolutionary unconstrained (dN/dS for *matR*  = 0.975, *matK*  = 0.427 compared with *rbcL*  = 0.074, *atpB*  = 0.064). Considering the differences in tempo of evolution between *matK* and *matR*, the relative decline in informativeness in *matK* at deep historic levels could be a function of elevation in multiple hits that could obscure phylogenetic signal, but nonstationarity may be another factor. The low rate of substitution in *matR* and its 2.3–15 fold dN/dS are expected to increase signal to noise ratios by curbing the overall proportions of multiple hits [80], [81]. This mode and tempo of evolution in *matR* may account for maintaining higher informativeness profiles at deeper history in the rosids (Figs. 5, 6).

The decline in the informativeness profile of *matK* ≥∼54 MY ago (Figure 5) corresponds to the epoch at which the problematic orders Myrtales, Geraniales and Crossosomatales diverged, possibly causing their uncertain placement in the rosids tree (Figure 2). In contrast, the decline in recent epochs in the *rbcL* and *atpB* informativeness (≤∼60 MY; Figure 5) may account for their low utility in discerning pattern of divergences for orders emerging at that evolutionary period (Figure 2). Notable is the considerably low standard deviation values for informativeness at deeper evolutionary history for all four genes (Figure 5). These values suggest minimum stochasticity in phylogenetic signal for the genes despite the substantial differences in their tempo and mode of evolution.

Phylogenetic informativeness should be considered alongside noise caused by homoplasy and multiple hits, particularly in rapidly evolving genes. Homoplasy (1-CI) for *matK*, *rbcL* and *atpB* values are quite comparable (0.626, 0.647 and 0.610). In contrast, *matR* deviates from these three genes by having substantially lower homoplasy, 0.308. This is possibly an outcome of a compounding effect of low rate of nucleotide substitution and high dN/dS ratio (0.975), which points to evenness in mutations across the three codon positions, an evolutionary mode that minimizes the potential of saturation and multiple hits. The homoplasy value for *matK*, being intermediate between the two slowly evolving genes *rbcL* and *atpB*, does not reflect the common notion that rapidly evolving genes are highly homoplasic.

### Tree robustness and accuracy

The question is how these varied informativeness profiles and homoplasy are translated into tree robustness. The *matK* MP strict consensus tree resolved 98% of the expected nodes (Table 1), approaching the full resolution achieved in the Wang et al. [19] multigene tree. This resolution is contrasted with 88%, 71%, and 80% in the *rbcL*, *atpB* and *matR* trees, respectively (Table 1). Bootstrap values for major nodes are much higher in *matK* compared with the other genes (Figure 3), with means and standard deviation being 99%±3 for *matK* vs. 63%±36, 74%±36, and 84%±28 for *rbcL*, *atpB*, and *matR*, respectively. Thus, not only notable differences in magnitude of resolution and support exist, but also a substantial variation in node support across the rosids for the three latter genes (Figure 1, 3).

The ultimate goal in phylogenetic reconstruction is accuracy in depicting true patterns of historic divergences. The Wang et al. [19] ML tree represents the best available hypothesis for the rosids phylogeny. Compared to this reference tree, the backbone of the *matK* ML tree displays one topological incongruence by depicting the phylogenetically difficult-to-place Geraniales and Crossosomatales sister to remaining rosids instead of being basal in a malvids clade, but BS support is <50% (Figure 2). Accuracy was reduced in the *matR* tree as a consequence of the inability to recover the fabid clade and in the placement of the COM clade, Zygophyllales, Myrtales, and Geraniales (Figure 2). These topological inconsistencies receive weak support (Figure 2). Homoplasy cannot account for the incongruences since *matR* displays the lowest degree of homoplasy among all four genes (Table 1). It is likely that such shortcomings are a consequence of the low signal in *matR* across the rosids tree (Figure 5, Table 1). Fong and Fujita [67] have shown in three data sets of vertebrates genes that phylogenetic signal was greatly reduced although they were less subject to homoplasy. Zhu et al. [48] recovered the same topology for the rosids in their *matR* partition, which they attributed to a difference in history or evolutionary phenomena for *matR*.

Accuracy varies between the slowly evolving/constrained *rbcL* and *atpB* ML trees (Figure 2), despite the comparable amounts of PI characters and net informativeness (Figure 4, 5, Table 1). The pronounced differences in topological accuracy between the *atpB* and *rbcL* trees and that of the *matK* cannot be explained by homoplasy since CI values of three genes are comparable, and that of *matK* being intermediate between *atpB* and *rbcL* (*atpB* = 0.390, *matK* = 0.374, *rbcL* = 0.353). Therefore, it follows that tree accuracy and robustness are impacted by disparities in phylogenetic signal. Net phylogenetic informativeness in *matK* is ≥3× of the slowly evolving genes (Table 1), and its phylogenetic informativeness consistently surpassed them across rosids evolution (Table 1, Figure 5). Further, the number of PI characters in *matK* is 2.2–2.4 times that of the other genes (Table 1).

### Phylogenetic informativeness at codons level

The informativeness profile of the 3^rd^ codon positions consistently superseded those of the 1^st^ and 2^nd^ in all genes across the rosids evolutionary history, but disparity in informativeness is inversely proportional to their respective dN/dS ratio (Table 1, Figure 6). The dN/dS for constrained *matR* (0.968) and *matK* (0.427) are 6–16 times the dN/dS of unconstrained *rbcL* (0.074) and *atpB* (0.062), reflecting the differential rates of substitution at the three codon positions. The high uniformity in informativeness profiles across codon positions for unconstrained *matR* and the 10 fold difference in net informativeness between the 3^rd^ codon position and the 2^nd^ of constrained *atpB* and *rbcL* reflect the two extremes in dN/dS ratios (Table 1). In *matK*, the 3^rd^ codon position is twice as informative as the 2^nd^, in concordance with its dN/dS ratio. In general, the 2^nd^ codon position exhibited the least amount of informativeness (Figure 6). Further, homoplasy was lowest in the 2^nd^ codon positions for all genes except for *matR* where it was comparable for 1^st^ and 2^nd^ codon positions. Nucleotide substitutions in the 2^nd^ codon position are translated into 100% nonsynonymous mutations [34], and consequently it is highly constrained evolutionarily. These findings are in agreement with the Björklund's [39] partitioned phylogenetic analyses of cytochrome *b* codon partitions in vertebrate where performance is lowest in 2^nd^ codon and highest in the 3^rd^.

Tree resolution in codon position partition analyses followed closely the inherent dN/dS for the genes. The 3^rd^ codon positions of constrained *rbcL* and *atpB* provided higher resolution than 1^st^+2^nd^ partition, whereas the amount of resolution was lower in the *matR* 3^rd^ codon position than the equally-informative 1^st^ and 2^nd^ codon positions combined. In *matK*, the amount of resolution was the same for the two partitions (Table 1). Similar patterns are notable when the number of PI characters are considered (Table 1). Strong correlation exists between phylogenetic informativeness and PI characters for the codon partitions (r^2^ = 0.933, F_1,12_ = 477, P<0.0001).

The higher rate of substation in the 3^rd^ codon position has been negatively construed since such an attribute is expected to increase the likelihood of site saturation and to elevate the degree of homoplasy [34]. Although this might be a cautionary points for genomic regions with excessively higher rates of substitution, such animal mitochondrial DNA, it has been overly generalized, leading to the tendency of excluding or down weighting of the 3^rd^ codon position in phylogenetic reconstruction, e.g. [7], [27], [29], [30], [32]. Simmons et al. [42] contended that PI characters of 3^rd^ codon position in *rbcL* and *atpB* angiosperms data set outperformed the 1^st^ and 2^nd^ combined in phylogenetic signal, and that Jackknife support was 14% higher with the 3^rd^ codon position compared with the tree based on 1^st^ and 2^nd^ combined in seed plants phylogenetic study. Imposing a 4∶17∶1 weighing criteria for the 1^st^, 2^nd^ and 3^rd^ codon positions in an analyses of cytochrome *b* [28] reduced resolution and increased probabilities of support for erroneous trees. Similarly, it has been found [82] that exclusion of the 3^rd^ codon position led to a substantial problem in recovering the true tree.

## Conclusions

Our study demonstrates that tree robustness and phylogenetic informativeness for the four genes work in concert with their mode and tempo of evolution. Phylogenetic signal from rapidly evolving and unconstrained *matK* provides by far the most structure and accuracy, whereas slowly evolving, constrained and unconstrained, genes display decreasing degrees of informativeness and tree structure. The 3^rd^ codon positions consistently supersede the 1^st^ and 2^nd^ positions in phylogenetic signal, and its differential informativeness is accentuated in the constrained genes. The study underscores the need for assessments of phylogenetic informativeness of genomic regions for a given biological lineage within the framework of overall rates of nucleotide as well as nonsynonymous substitutions across their historic divergence. A priori judgments on performances of genomic regions without empirical data may hinder efforts aiming at achieving the best phylogenetic hypothesis. Specifically, our findings in the rosids argues against the notion that arbitrarily discourages the use of rapidly evolving genomic regions in deep phylogenetics due to potential multiple hits, homoplasy and saturation [9], [11], [14], [15], [71], [83]. Simmons et al. [43] demonstrated that increasing rates of evolution in a simulation model consistently improved resolution. Yang [2] concluded that optimal limits for sequence divergence are higher than previously suggested for saturation of substitutions and, consequently, the problem of saturation may have been exaggerated. In a phylogenetic analyses of an *rbcL* data set for green plants, Källersjö et al. [10] asserted that homoplasy can provide phylogenetic structure. We have demonstrated in a study of early diverging angiosperms [25] that a PI site for *matK* provides more structure than that of *rbcL*, and that homoplasy in *matK* has less negative impact on phylogenetic structure than it does in *rbcL*. In a phylogenetic analysis of 1070 genes in a yeast data set, Salichos and Rokas [84] found that using slowly evolving genes and conserved sites increased incongruence across many internodes. Recently, Magallón et al. [84] demonstrated in an assessment of land plant phylogeny that *matK* provides phylogenetic signal and structure matching those derived from a concatenated, three slowly evolving genes data.

The per-site informativeness profiles, which excludes gene length bias, mirrors closely their corresponding net informativeness profiles and the two are highly correlated (r^2^ = 0.903, F_1,3_ = 19, P<0.0499). The number of nucleotides sequenced for *matR* (1822 nucleotides) exceeds those of *matK* (1672), *atpB* (1496) and *rbcL* (1427). Nevertheless, *matR* is at disadvantage in terms of number of PI characters and net-informativeness and, thus, lessening its cost-effectiveness per nucleotide sequenced. The *matK* gene stands at the other end of the cost-effectiveness spectrum.

Although the study promotes the consideration of rapidly evolving regions in phylogenetic reconstruction, homology assessment of sequence alignments at deep histories should not be compromised since this step represents a crucial foundation in molecular phylogenetics. The rosids divergence and diversification spans some 108 million years, and thus it would be useful to carry out similar detailed studies for groups with substantially deeper evolutionary histories.

## Supporting Information

Figure S1 Detailed *matK* RAxMl tree for the rosids and representatives of remaining core eudicots.
(PDF)

Figure S2 Detailed *matR* RAxMl tree for the rosids and representatives of remaining core eudicots.
(PDF)

Figure S3 Detailed *rbcL* RAxMl tree for the rosids and representatives of remaining core eudicots.
(PDF)

Figure S4 Detailed *atpB* RAxMl tree for the rosids and representatives of remaining core eudicots.
(PDF)

Table S1 Taxa used in this study. The species used, their family and order affiliation and the GenBank accessions numbers.
(PDF)

Table S2 Bootstrap support for rosids orders represented by more than one taxon.
(DOCX)
