# Supplementary figures and images for: Impact of Gene Molecular Evolution on Phylogenetic Reconstruction: A Case Study in the Rosids (Superorder Rosanae, Angiosperms)
Source: PLoS One. 2014 Jun 16;9(6):e99725. doi: 10.1371/journal.pone.0099725 (PMC4059714; doi:10.1371/journal.pone.0099725)

Figure S1

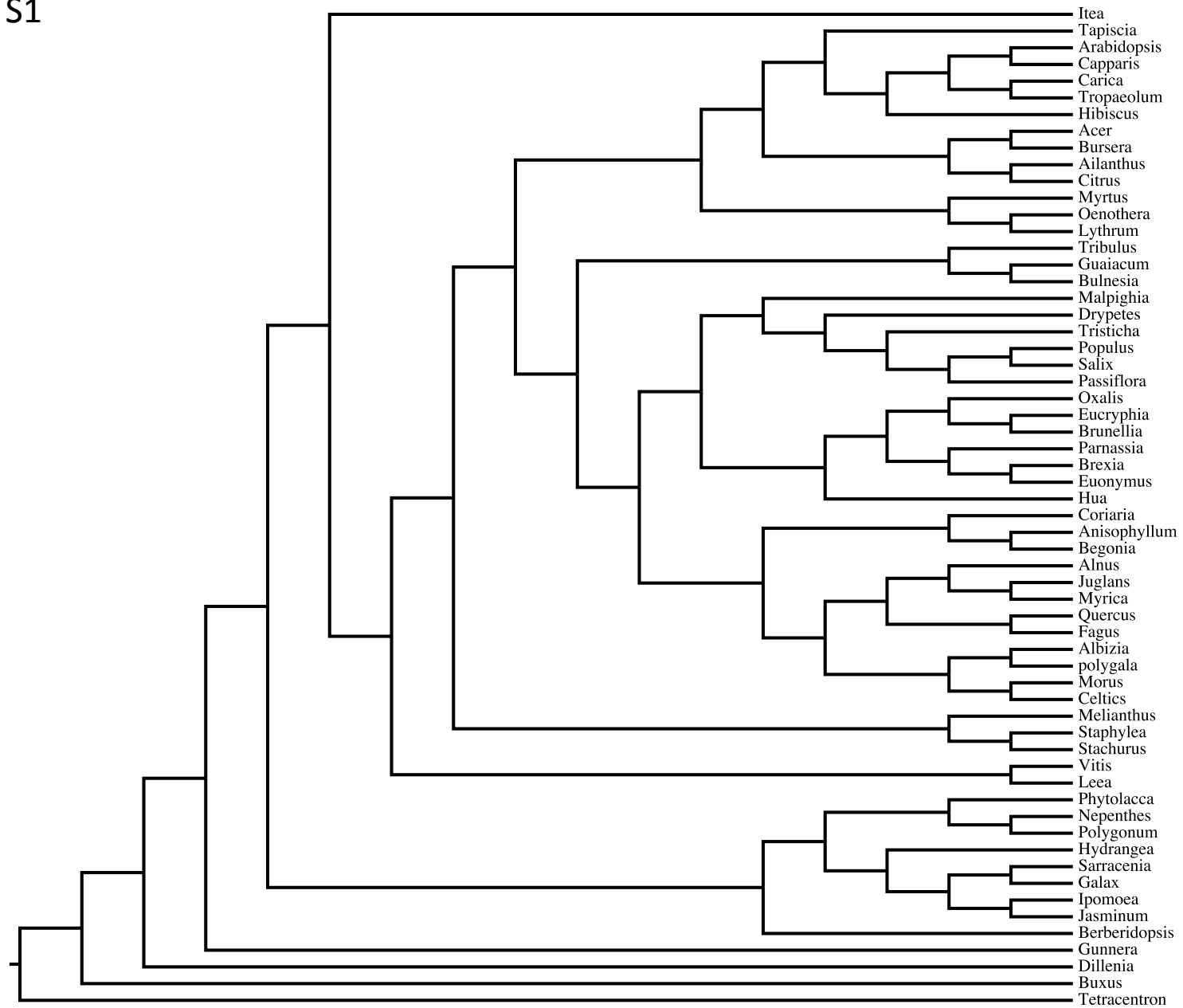

Supplement: Figure S1 — Detailed matK RAxMl tree for the rosids and representatives of remaining core eudicots. (PDF) [file pone.0099725.s001.pdf]

Figure S2

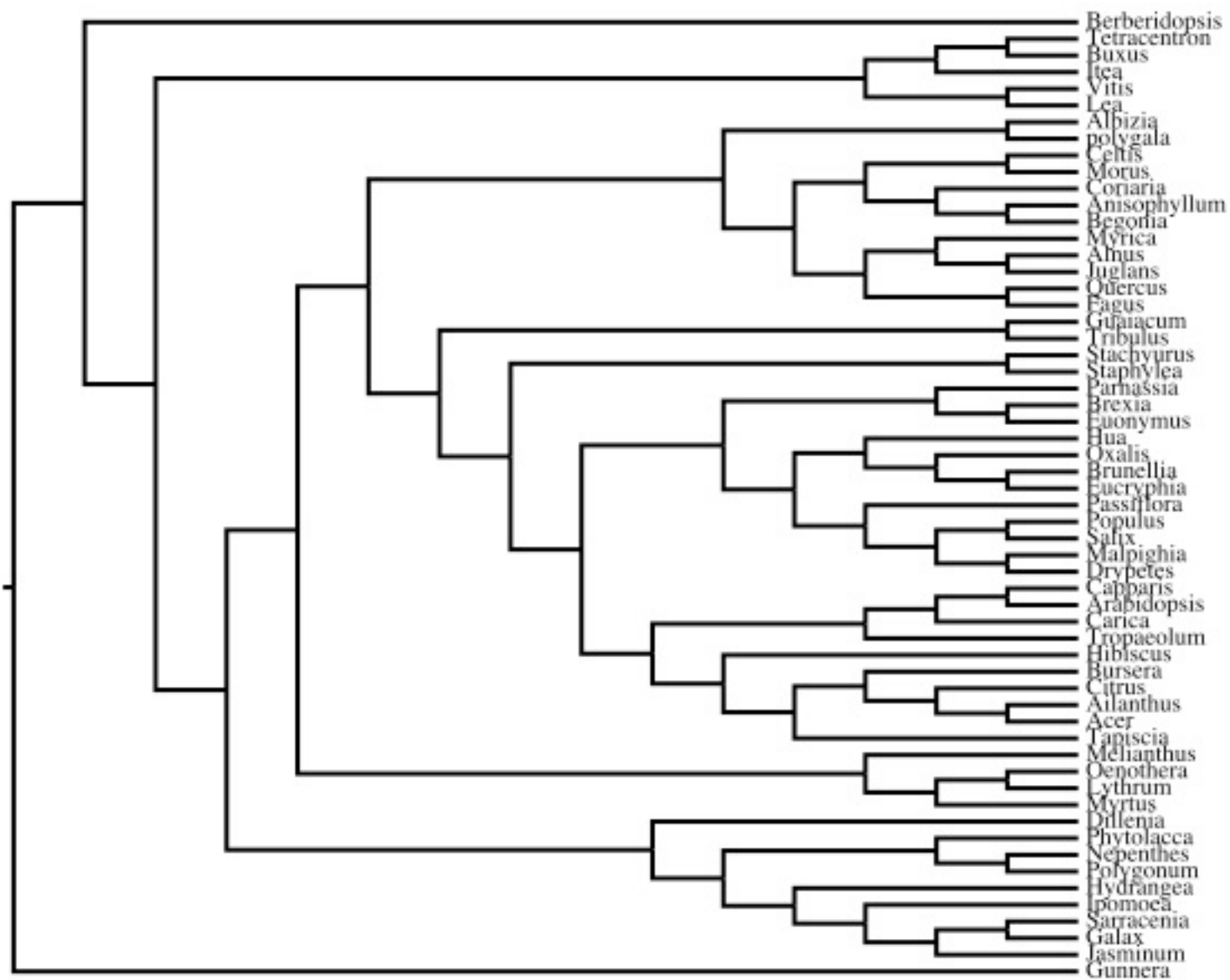

Supplement: Figure S2 — Detailed matR RAxMl tree for the rosids and representatives of remaining core eudicots. (PDF) [file pone.0099725.s002.pdf]

Figure S3

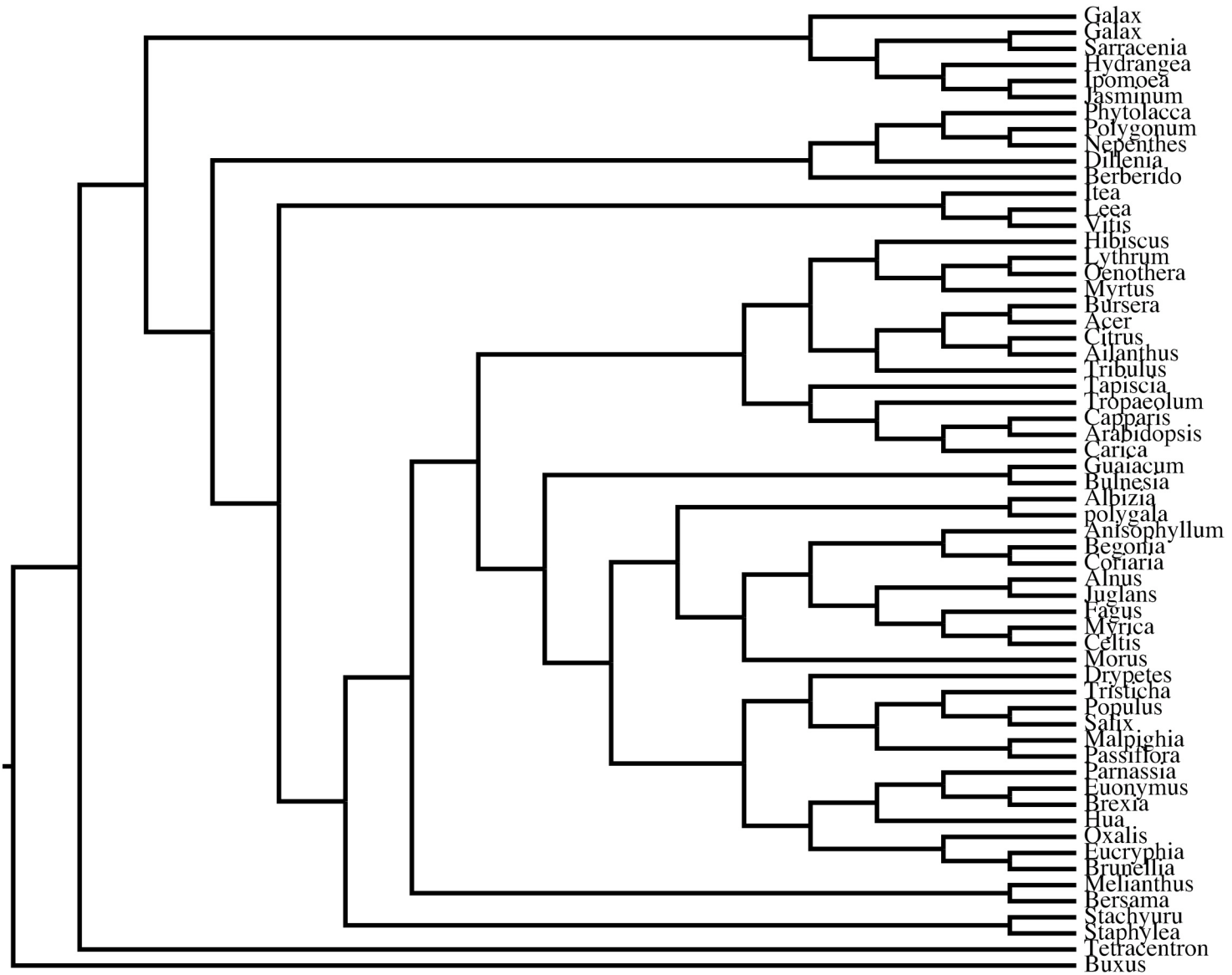

Supplement: Figure S3 — Detailed rbcL RAxMl tree for the rosids and representatives of remaining core eudicots. (PDF) [file pone.0099725.s003.pdf]

Figure S4

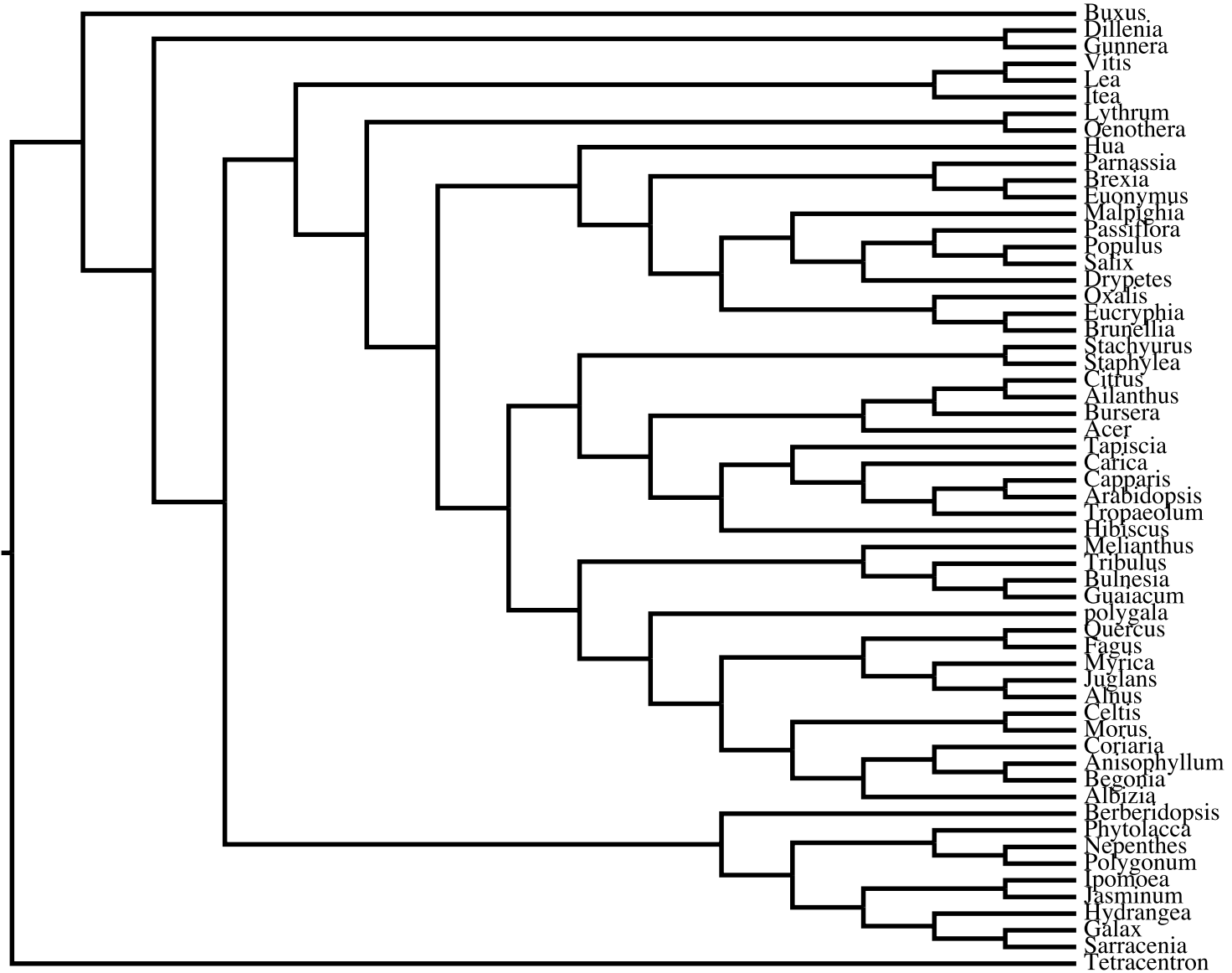

Supplement: Figure S4 — Detailed atpB RAxMl tree for the rosids and representatives of remaining core eudicots. (PDF) [file pone.0099725.s004.pdf]
