# Supplementary material for: Impact of Gene Molecular Evolution on Phylogenetic Reconstruction: A Case Study in the Rosids (Superorder Rosanae, Angiosperms)
Source: PLoS One. 2014 Jun 16;9(6):e99725. doi: 10.1371/journal.pone.0099725 (PMC4059714; doi:10.1371/journal.pone.0099725)
Supplement: Table S1 — Taxa used in this study. The species used, their family and order affiliation and the GenBank accessions numbers. (PDF) [file pone.0099725.s005.pdf]

Table S1

Order	Family	Species/matK	Sequences /matK	Source of material	Species/rbcL	Sequences/rbcL	Species/atpB	Sequences/atpB	Species/matR	Sequences/matR
Berberidopsidales	Berberidopsidaceae	*Berberidopsis corralina*	AY042554; KF224974	Qiu 97042	Berberidopsis corralina	EU002274	*Berberidopsis corralina*	EU002158	*Berberidopsis beckleri*	DQ401394
Brassicales	Brassicaceae	*Arabidopsis thaliana*	NC_000932		Arabidopsis thaliana	NC_000932	*Arabidopsis thaliana*	NC_000932	*Arabidopsis thaliana*	EU999002
Brassicales	Capparidaceae	*Capparis hastata*	AY483228		Capparis flexuosa	M95754	*Capparis spinosa*	AF035900	*Capparis membranifolia*	AF520146
Brassicales	Caricaceae	*Carica papaya*	AY483221		Carica papaya	M95671	*Carica papaya*	NC_010323	*Carica papaya*	NC_010323
Brassicales	Tropaeolaceae	*Tropaeolum majus*	AY483224		Tropaeolum majus	L14706	*Tropaeolum tricolor*	AF035917	*Tropaeolum majus*	AF520137
Buxales	Buxaceae	*Buxus sempervirens*	AF543728		Buxus microphylla	NC_009599	*Buxus microphylla*	NC_009599	*Buxus sempervirens*	AF197786
Caryophyllales	Nepenthaceae	*Nepenthes edwardsiana*	DQ840248		Nepenthes alata	L01936	*Nepenthes alata*	AF093388	*Nepenthes sp.*	DQ401379
Caryophyllales	Phytolaccaceae	*Phytolacca americana*	AY042631		Phytolacca americana	M62567	*Phytolacca americana*	AF528855	*Phytolacca americana*	DQ401389
Caryophyllales	Polygonaceae	*Polygonum cespitosum*	KF224975	Hinkle 376 (VPI)	Polygonum cuspidatum	AB019031	*Polygonum sachalinense*	AJ235569	*Polygonum sp.*	DQ401388
Celastrales	Celastraceae	*Brexia madagascariensis*	AY935899		Brexia madagascariensis	L11176	*Brexia madagascariensis*	AJ235419	*Brexia madagascariensis*	AY674482
Celastrales	Celastraceae	*Euonymus americanus*	KF224976	A. Hinkle 338 (VPI)	Euonymus americanus	EU002277	*Euonymus americanus*	EU002160	*Euonymus alatus*	AY674511
Celastrales	Parnassiaceae	*Parnassia fimbriata*	AY935911		Parnassia fimbriata	L01939	*Parnassia glauca*	AY935835	*Parnassia palustris*	AY121491
Cornales	Hydrangeaceae	*Hydrangea macrophylla*	AB236030		Hydrangea sp.	AY725856	*Hydrangea arborescens*	AF093381	*Hydrangea macrophylla*	AY453091
Crossosomatales	Stachyuraceae	*Stachyurus praecox*	DQ443457		Stachyurus retusus	DQ307097	*Stachyurus praecox*	AJ235609	*Stachyurus chinensis*	AY121489
Crossosomatales	Stachyuraceae	*Staphylea trifolia*	GU266607		Staphylea trifolia	AY646111	*Staphylea colchica*	EU002168	*Staphylea trifolia*	AF520105
Cucurbitales	Anisophylleaceae	*Anisophyllea sororia*	AY968444		Anisophyllea sororia	AY973487	*Anisophyllea sororia*	AY973440	*Anisophyllea manausensis*	AY121498
Cucurbitales	Cucurbitaceae	*Begonia oxyloba*	AY968445		Begonia glabra	AF008962	*Begonia oxyloba*	AY968426	*Begonia laciniata*	AY121500
Cucurbitales	Coriariaceae	*Coriaria sarmentosa*	AB016460		Coriaria sarmentosa	AF149000	*Coriaria myrtifolia*	AJ235443	*Coriaria nepalensis*	AY121501
Dilleniales	Dilleniaceae	*Dillenia indica*	KF224977	M.J. Moore s.n.; cult at Univ. of Florida	Dillenia indica	L01903	*Dillenia retusa*	AF095732	*Dillenia indica*	AF520095
Ericales	Diapensiaceae	*Galax urceolata*	L48576; KF224986	Hinkle 330 (VPI)	Galax aphylla	Z80184	*Galax urceolata*	AY725936	*Galax urceolata*	AF421007
Ericales	Sarraceniaceae	*Sarracenia leucophylla*	GU266611		Sarracenia flava	L01952	*Sarracenia flava*	AJ235594	*Sarracenia flava*	AF421028
Fabales	Fabaceae	*Albizia julibrissin*	AY386855		Albizia saman	Z70149	*Albizia julibrissin*	AF209524	*Albizia schimperiana*	AF520168
Fabales	Polygalaceae	*Polygala californica*	AY386842		Polygala cruciata	L01945	*Polygala cruicata*	AJ235568	*Polygala paucifolia*	AY453080
Fagales	Betulaceae	*Alnus firma*	AB060053		Alnus incana	X56618	*Alnus sinuata*	AY147101	*Alnus henryi*	AF520062
Fagales	Fagaceae	*Fagus crenata*	AB046500		Fagus crenata	AB060567	*Fagus grandifolia*	AY935855	*Fagus engleriana*	AF520082
Fagales	Juglandaceae	*Juglans nigra*	AF118036		Juglans nigra	U00437	*Juglans nigra*	AF209609	*Juglans mandshurica*	AF520073
Fagales	Myricaceae	*Myrica gale*	AY191715		Myrica gale	X69530	*Myrica cerifera*	AJ235537	*Myrica gale*	DQ110347
Fagales	Fagaceae	*Quercus gemelliflora*	KF224984		Quercus gemelliflora	AB125019	*Quercus nigra*	EU002167	*Quercus rubra*	DQ110356
Geraniales	Melianthaceae	*Melianthus major*	GU266598		Melianthus	AJ403027	*Melianthus major*	AJ235532	*Melianthus major*	AY453087
Gunnerales	Gunneraceae	*Gunnera manicata*	AY042596		Gunnera manicata	EU002279	*Gunnera manicata*	EU002162	*Gunnera monoica*	DQ401383
Huerteales	Tapisciaceae	*Tapiscia sinensis*	KF224978	Chase 1201 (K)	Tapiscia sinensis	AY646112	*Tapiscia sinensis*	AF209685	*Tapiscia sinensis*	AF520103
Lamiales	Oleaceae	*Jasminum nudiflorum*	NC_008407		Jasminum nudiflorum	DQ673255	*Jasminum nudiflorum*	NC_008407	*Jasminum abyssinicum*	AF520152
Malpighiales	Putranjivaceae	*Drypetes madagascariensis*	AY552457		Drypetes roxburghii	M95757	*Drypetes littoralis*	AB233718	*Drypetes perreticulata*	AF520185
Malpighiales	Malpighiaceae	*Malpighia emarginata*	AF344561		Malpighia coccigera	AJ235784	*Malpighia glabra*	AB233692	*Malpighia glauca*	AF520187
Malpighiales	Passifloraceae	*Passiflora suberosa*	GU266608		Passiflora biflora	EU017122	*Passiflora biflora*	EU017086	*Passiflora edulis*	AF520188
Malpighiales	Salicaceae	*Populus alba*	NC_008235		Populus alba	NC_008235	*Populus alba*	NC_008235	*Populus maximowiczii*	AY674556
Malpighiales	Salicaceae	*Salix eriocephala*	AY669058		Salix paradoxa	AJ418840	*Salix reticulata*	AJ235590	*Salix raddeana*	AF520191
Malpighiales	Podostemaceae	*Tristicha trifaria*	AB113745		Tristicha trifaria	AB113755	*Tristicha*		*Tristicha trifaria*	HQ331834
Malvales	Malvaceae	*Hibiscus macrophyllus*	AB181100		Hibiscus punaluuensis	AJ233121	*Hibiscus punaluuensis*	AJ233064	*Hibiscus rosa-sinensis*	DQ110343
Myrtales	Lythraceae	*Lythrum hyssopifolia*	KF224979	D. Soltis 2704, Dept Botany greenhouse	Lythrum hyssopifolia	L10218	*Lythrum salicaria*	AF209621	*Lythrum salicaria*	AF520110
Myrtales	Myrtaceae	*Myrtus communis*	AY525136		Myrtus communis	AF294254	*Myrtus communis*	JF268426.1	*Myrtus communis*	EU281109
Myrtales	Onagraceae	*Oenothera elata subsp. Hookeri*	NC_002693		Oenothera elata subsp. H	NC_002693	*Oenothera elata*	NC_002693	*Oenothera biennis*	AY453083
Oxalidales	Brunelliaceae	*Brunellia oliveri*	AY935926		Brunellia oliveri	AF291938	*Brunellia sp.*	AY935850	*Brunellia acutangula*	DQ110330
Oxalidales	Cuunoniaceae	*Eucryphia cordifolia*	KF224980	Soltis & Soltis 74.0135	Eucryphia cordifolia	AF291931	*Eucryphia milliganii*	AJ235470	*Eucryphia milliganii*	AY674510
Oxalidales	Huaceae	*Hua gabonii*	AY935903		Hua gabonii	AY935726	*Hua gabonii*	AY935830	*Hua gabonii*	AY121490
Oxalidales	Oxalidaceae	*Oxalis latifolia*	KF224983		Oxalis latifolia	EU002282	*Oxalis latifolia*	EU002165	*Oxalis corymbosa*	AF520198
Rosales	Ulmaceae	*Celtis yunnanensis*	AY263925		Celtis yunnanensis	L12638	*Celtis philippensis*	AY263961	*Celtis bungeana*	AF520086
Rosales	Moraceae	*Morus indica*	NC_008359		Morus indica	NC_008359	*Morus indica*	NC_008359	*Morus alba*	AY453084

Sapindales	Sapindaceae	*Acer campestre*	AJ438795		Acer fabri	EF186772	*Acer saccharum*	AF035893	*Acer mono*	AF520112
Sapindales	Simaroubaceae	*Ailanthus altissima*	AY128208		Ailanthus altissima	L12566	*Ailanthus triphysa*	EU042774	*Ailanthus altissima*	AF520106
Sapindales	Burseraceae	*Bursera fagaroides*	KF224981	A. Hinkle 369 (VPI)	Bursera inaguensis	L01890	*Bursera inaguensis*	AF035899	*Bursera simaruba*	EU281100
Sapindales	Rutaceae	*Citrus sinensis*	NC_008334		Citrus sinensis	NC_008334	*Citrus sinensis*	NC_008334	*Citrus sinensis*	AY453100
Saxifragales	Iteaceae	*Itea virginica*	AF274618; KF224987	Hinkle 327 (VPI)	itea virginica	L11188	*Itea ilicifolia*	AF093383	*Itea yunnanensis*	AF520099
Solanales	Convolvulaceae	*Ipomoea purpurea*	GU266612		Ipomoea purpurea	NC_009808	*Ipomoea purpurea*	NC_009808	*Ipomoea alba*	EU281111
Trochodendrales	Trochodendraceae	*Tetracentron sinense*	AF274633; KF224988	Qiu 90009 NCU	Tetracentron sinense	L12668	*Tetracentron sinense*	AF093422	*Tetracentron sinense*	AF197791
Vitales	Vitaceae	*Leea guineensis*	AF274621		Leea guineensis	AJ235783	*Leea guineensis*	AJ235520	*Leea guineensis*	AY674530
Vitales	Vitaceae	*Vitis aestivalis*	NC_007957		Vitis aestivalis	L01960	*Vitis vinifera*	NC_007957	*Vitis riparia*	AY453123
Zygophyllales	Zygophyllaceae	*Bulnesia arborea*	EU002172		Bulnesia arborea	EU002275	*Bulnesia arborea*	EU002159	*Bulnesia*	
Zygophyllales	Zygophyllaceae	*Guaiacum sanctum*	KF224982	NCU:Chase 133	Guaiacum sanctum	AJ131770	*Guaiacum sanctum*	AJ235486	*Guaiacum sanctum*	AY674517
Zygophyllales	Zygophyllaceae	*Tribulus terrestris*	KF224985	VPI 94656	Tribulus terrestris	DQ267165	*Tribulus terrestris*	GQ220747	*Tribulus terrestris*	AF520097
