# Supplementary material for: Impact of Gene Molecular Evolution on Phylogenetic Reconstruction: A Case Study in the Rosids (Superorder Rosanae, Angiosperms)
Source: PLoS One. 2014 Jun 16;9(6):e99725. doi: 10.1371/journal.pone.0099725 (PMC4059714; doi:10.1371/journal.pone.0099725)
Supplement: Table S2 — Bootstrap support for rosids orders represented by more than one taxon. (DOCX) [file pone.0099725.s006.docx]

Table S2.

| Orders | *matK* | *matR* | atpB | *rbcL* |
| --- | --- | --- | --- | --- |
| Myrtales | 100 | 100 | 99 | 100 |
| Crossosomatales | 100 | 100 | 100 | 98 |
| Cucurbitales | 100 | 100 | 98 | 89 |
| Rosales | 100 | 98 | 100 | na |
| Fagales | 100 | 100 | 97 | 99 |
| Fabales | 100 | 95 | na | 78 |
| Oxalidales | 100 | 100 | 82 | 99 |
| Celastrales | 100 | 100 | 100 | 100 |
| Malpighiales | 100 | 100 | 99 | 67 |
| Zygophyllales | 100 | 100 | 89 | 100 |
| Sapindales | 100 | 97 | 97 | 58 |
| Brassicales | 100 | 97 | 96 | 75 |

na: not applicable due to lack of monophyly
